# Generation of *Fusarium oxysporum*-suppressive soil with non-soil carriers using a multiple-parallel-mineralization technique

**DOI:** 10.1038/s41598-022-10667-1

**Published:** 2022-05-13

**Authors:** Jamjan Meeboon, Akinori Ando, Jun Ogawa, Kenji Miyamoto, Yasuo Kato, Makoto Shinohara

**Affiliations:** 1 Institute of Vegetable and Floriculture Science, National Agriculture and Food Research Organization (NARO), 360 Ano, Tsu, Mie 514-2392 Japan; 2 Division of Applied Life Sciences, Graduate School of Agriculture, Kyoto University, Kyoto, Japan; 3 Research Unit for Physiological Chemistry, Kyoto University, Kyoto, Japan; 4 Department of Biosciences and Informatics, Keio University, Yokohama, Japan; 5 Biotechnology Research Center and Department of Biotechnology, Toyama Prefectural University, Imizu, Japan

**Keywords:** Biotechnology, Developmental biology

## Abstract

Disease-suppressive soils exist worldwide. However, the disease-suppression mechanism is unknown, and it’s unclear how to produce such soils. The microbiota that develop in a multiple-parallel-mineralization system (MPM) can increase nutrient production efficiency and decrease root disease in hydroponic systems. Artificial media inoculated with MPM microorganisms can degrade organic matter to produce inorganic nutrients similarly to natural soil, but it’s unknown whether they can also suppress pathogen growth. Here, we produced an artificial medium that inhibited root disease similarly to disease-suppressive soils. Microbial MPM culture solution was inoculated into non-soil carriers (rockwool, rice husk charcoal, and vermiculite) to test whether it could suppress growth of *Fusarium oxysporum* f. sp. *lactucae* J. C. Hubb. & Gerik. We inoculated *F. oxysporum* f. sp. *conglutinans* (Wollenweber) Snyder et Hansen strain Cong:11 and *F. oxysporum* f. sp. *lactucae* J. C. Hubb. & Gerik into artificial media sown each with *Arabidopsis thaliana* (L.) Heynh. and *Lactuca sativa* L. var. *capitata* supplemented with MPM culture microbes. The MPM microorganisms suppressed *F. oxysporum* f. sp. *lactucae* J. C. Hubb. & Gerik growth and prevented plant disease. Thus, MPM-inoculated non-soil carriers that can generate inorganic nutrients from organic matter may also suppress disease in the absence of natural soil. Our study shows novel creation of a disease-suppressive effect in non-soil media using the microbial community from MPM culture solution.

## Introduction

Disease-suppressive soils have been recognized for more than 60 years^1^ . Hypotheses to explain the disease-suppression mechanisms have been based on differences in the soil minerals, antagonistic microorganisms, and microbial ecosystem, but the actual mechanism is unknown^1–4^ . Previous attempts to generate disease-suppressive soil through inoculation with antagonistic microbes that suppress or out-compete the pathogens have been ineffective, since the microbes could not become established within the soil^5^. To establish these antagonistic microorganisms, it’s necessary to reconstruct or simulate natural soil microbial ecosystems, but the ability of natural soil to degrade organic matter into inorganic nutrients has not been artificially reproduced. Achieving this ability would be an important breakthrough because of problems such as eutrophication caused by inorganic fertilizers and their high cost, as well as the growing availability of organic wastes that should be recycled in agriculture rather than discarded. In the soil, degradation of organic nitrogen proceeds in two steps: ammonification and nitrification^.^ Nitrogen-cycling bacteria (which produces nitrates) are inactivated by exposure to high concentrations of organic matter^6^ . When these bacteria are inactivated, degradation of organic matter proceeds only until ammonia is produced, which is problematic, because many plants prefer nitrates, and this results in poor growth.

Shinohara et al. (2011) developed a novel method to efficiently generate nitrate in non-soil media, such as in the water used in hydroponic systems. In summary, the method consists of three steps: (1) add 10 g/L of soil to water as a source of microbial inoculum, (2) add small amounts of organic fertilizer, and (3) aerate the solution. The resulting system can degrade organic matter into inorganic nutrients such as nitrate by enabling simultaneous ammonification and nitrification. We call this the “multiple-parallel-mineralization” (MPM) method. Hydroponic conditions with no solid medium, cultivation with MPM solution suppressed root diseases such as bacterial wilt disease and Fusarium wilt^2,3,7^ . When the MPM culture solution is inoculated into a non-soil carrier, the carrier acquires the ability of a natural soil to degrade organic matter into inorganic nutrients^8^. This non-soil medium may also suppress the growth of pathogenic organisms such as *Fusarium* species^9^. To test this possibility, we examined whether immobilizing the MPM microorganisms on a non-soil solid carrier could achieve the same effect as a disease-suppressing soil. (Here, *immobilizing* means that the microorganisms remain within the carrier rather than being lost by leaching when the solution is refreshed). *Arabidopsis thaliana* (L.) Heynh. and *Lactuca sativa* L. var. *capitata* are great hosts each for *Fusarium oxysporum* f. sp. *conglutinans* (Wollenweber) Snyder et Hansen strain Cong:11 and *F. oxysporum* f. sp. *lactucae* J. C. Hubb. & Gerik^7,10–12^. To the best of our knowledge, this is the first study that successfully developed a reproducible disease-suppressive ecosystem using the MPM system.

## Materials and methods

### MPM solution preparation

We prepared the MPM solution^7^ as follows: we added 1 g of nursery soil (Nae-ichiban; Sumirin Agro-Products, Aichi, Japan) and 1 g/L fish-based soluble fertilizer (Yaizu Suisankagaku Industry, Yaizu, Japan) to 100 mL of distilled water in a flask, and then incubated the flask for 2 weeks in the dark at 25 °C in an incubator (CN-25C; Mitsubishi Electric, Tokyo, Japan) with shaking (120 rpm). We measured the inorganic N concentrations daily using Reflectoquant test strips (Merck, Darmstadt, Germany) for ammonium (catalogue number 116892), nitrite (no. 116973), and nitrate (no. 116971)^6^. We added and mixed the supplied reagents according to the manufacturer’s instructions for each kit, submerged the test strip, and measured the degree of color development using a reflective photometer (RQ-flex plus; Merck, Frankfurt, Germany). We then used the MPM solution as the microbial inoculum.

### Immobilization of MPM microorganisms on rockwool

We added 10 μL of the MPM solution and 1 mL of 1 g/L Yaizu fish-based soluble fertilizer to 1-cm^3^ Growcube rockwool cubes (Grodan, Roemond, the Netherlands) and incubated the cubes at 25 °C for 24 h in the dark. To investigate the effect of excess organic matter on the production of inorganic N, we added 1 mL of 1 g/L fish fertilizer diluted with distilled water per 1 cm^3^ of rockwool cube daily. After overnight incubation in the dark, each cube was rinsed with 1 mL of sterilized water, then after nitrate measurement, we repeated the addition of fish fertilizer and rinsing with sterilized water daily for 2 weeks. The inorganic N, ammonium, nitrite, and nitrate contents in the leachate were measured daily. We performed three independent experiments, each with one replicate, using this approach.

### Pathogen and culture conditions

We cultured the phytopathogenic fungus *Fusarium oxysporum* f. sp. *conglutinans* (Wollenweber) Snyder et Hansen strain Cong:11^7^ and *F. oxysporum* f. sp. *lactucae* J. C. Hubb. & Gerik strain H111^3^ in Petri dishes that contained 39 g/L potato dextrose agar (Difco, Detroit, MI, USA) at 4 °C in the dark. We then inoculated 3-mm-diameter agar plugs into 100 mL of 24 g/L potato dextrose broth (Difco) in a flask and cultured the solution on an orbital shaker (120 rpm) at 25 °C for 5 days in the dark. We filtered the culture through two layers of sterilized Prowipe (195 mm × 125 mm; Elleair, Tokyo, Japan) to eliminate elongated hyphae but to retain the microconidia, which we collected. We centrifuged the filtrate at 4000×*g* for 10 min and recovered the precipitate, which contained the microconidia. We then recovered and resuspended the microconidia by washing the precipitate three times with 50 mL of sterile distilled water and repeating the centrifugation. We adjusted the final concentration of the microconidial suspension to 1 × 10^3^ cells/mL by counting them under a microscope. We used Komada’s selective medium (1.0 g/L Na_2_B_4_O_7_·10 H_2_O, 1.0 g/L K_2_HPO_4_, 0.5 g/L KCl, 0.5 g/L MgSO_4_·7 H_2_O, 0.01 g/L Fe-Na-EDTA, 2.0 g/L L-asparagine, 20.0 g/L d-galactose, 15.0 g/L agar, 1.0 g/L pentachloronitrobenzene, 0.5 g/L sodium cholate, and 0.3 g/L streptomycin sulfate) as the selective medium for *F. oxysporum*^13^.

### Suppression of pathogen growth on multiple carriers

To investigate whether microorganisms from the MPM solution can suppress the growth of *F. oxysporum* f. sp. *lactucae* J. C. Hubb. & Gerik, we added MPM solution (10 µL) and 1 mL of sterilized Yaizu fish-based soluble fertilizer (1 g/L) to 1-cm^3^ sterilized rockwool cubes. We inoculated the cubes with MPM solution (50 µL) and added 5 mL of sterilized fertilizer (1 g/L), and repeated this inoculation using two other non-soil media: rice husk charcoal (about 5 cm^3^) and vermiculite (about 5 cm^3^). We incubated the inoculated media in the dark at 25 °C for 3 days, using uninoculated carriers as the control. After incubation, we extracted the microconidia as in “Pathogen and culture conditions” and then added 10 µL of the microconidial suspension per cm^3^ to the three media. Finally, we incubated the media in the dark at 25 °C for 7 days, with three replicates each. At the end of this period, we ground samples of the carrier (about 1 cm^3^ of rockwool and 5 cm^3^ of rice husk charcoal and vermiculite) in a sterilized mortar and pestle and diluted the rockwool samples with 9 mL of sterilized water to 10% of the original concentration and the other media with 5 mL of sterilized water to 50% of the original concentration. We determined the pathogen density by means of serial plating on Komada’s selective medium.

### Suppression of pathogen growth using different fertilizers

We tested the inhibition of *F. oxysporum* f. sp. *lactucae* J. C. Hubb. & Gerik growth by the microorganisms isolated from MPM culture with Yaizu fish-based soluble fertilizer, corn steep solids (Sigma-Aldrich, MO, USA), nutrient broth (Bacto; Becton Dickinson, Le Pont de Claix, France), Bacto peptone, Bacto tryptone, and Bacto yeast extract. We inoculated the carriers with MPM solution (10 µL), and added 1 mL of sterilized fertilizer (1 g/L) per cm^3^ of sterilized rockwool cubes, followed by incubation in the dark at 25 °C for 3 days, using uninoculated carriers as the control. After 3 days’ incubation, we inoculated the media with 10 µL of microconidia suspension per cm^3^. The carrier (1 cm^3^ of rockwool) was then ground with a sterilized mortar and pestle and diluted with 9 mL of sterilized water to produce a solution at 10% of the original concentration. We determined the pathogen density by means of sequential plating on Komada’s selective medium. We performed three independent experiments, each with one replicate, using this approach.

### Cultivation of *Arabidopsis thaliana *(L.) Heynh. and *Lactuca sativa* L. var. *capitata* on rockwool cubes

We used *Arabidopsis thaliana* (L.) Heynh. (Col-0) (Funakoshi, Tokyo, Japan) and *Lactuca sativa* L. var. *capitata* ‘Santa Clara’ (saladana lettuce; Tohoku Seed Co. Ltd., Utsunomiya, Tochigi, Japan) for the pathogen inoculation test. Seeds were sterilized at 70 °C under dry heat in a ventilated WFO-600ND oven (Eyela, Tokyo Rikakikai Co., Ltd., Tokyo, Japan) for 3 days, then soaked in 70% ethanol for 20 min, then in 5% sodium hypochlorite for 20 min. The sterilized seeds were then washed three times in sterilized distilled water. We prepared 100 mL of the inorganic nutrient solution using inorganic fertilizers (0.34 g/L of Otsuka House No. 1 and 0.23 g/L of Otsuka House No. 2; Otsuka Chemical, Osaka, Japan) to supply 60 mg/L of nitrate–N, and packed 100 cm^3^ of the rockwool cubes into 9-cm-diameter polypots (Tokai Kasei, Mino, Gifu, Japan). We then inoculated the rockwool with 1 mL of MPM solution that contained 60 mg/L of nitrate–N, added 100 mL of 1 g/L Yaizu fish-based soluble fertilizer or the inorganic nutrient solution, and incubated the media at 25 °C for 2 weeks in the dark. After incubation, we sowed one seed of lettuce or *A. thaliana* (L.) Heynh. per pot and incubated the pots in a growth chamber (Panasonic MLR-352, Tokyo, Japan) at 25 °C with a 12-h photoperiod (Light step 5:100 mV) for 10 days. There were three replicates (each representing one pot with one seedling) in each treatment. We conducted these experiments in accordance with relevant institutional, national, and international guidelines and legislation. All plant seeds used were commercially available.

### Pathogen inoculation

We inoculated 10-day-old seedlings of *A. thaliana* (L.) Heynh. and *L. sativa* in pots with a microconidial suspension of *F. oxysporum* f. sp. *conglutinans* (Wollenweber) Snyder et Hansen strain Cong:11 and *F. oxysporum* f. sp. *lactucae* J. C. Hubb. & Gerik (final density 1 × 10^2^ cells/mL in 100 cm^3^ of rockwool), respectively. All inoculated plants were grown in a growth chamber at 25 °C with a 12-h photoperiod (using LS5 lights) for 10 days. Uninoculated seedlings sown on rockwool with Yaizu fish-based soluble fertilizer were used as the control. There were three replicates (each representing one pot with one seedling) in each treatment.

## Results

### MPM solution preparation

We detected 250 to 350 mg/L of nitrate in the MPM solution cultured in flasks. This indicates that the microbial ecosystem in the MPM solution can degrade the organic N in the fish-based soluble fertilizer into inorganic nutrients.

### Immobilization of MPM microorganisms on rockwool

After incubation of the rockwool inoculated with MPM solution, each cube was rinsed daily with sterilized water. We detected nitrate in the effluent at up to 300 mg/L on the fourth day of incubation. This indicates that the microbial ecosystem in the rockwool has become capable of degrading fish-based soluble fertilizer into inorganic nutrients such as nitrate, as in natural soils, even when the ecosystem occupies artificial carriers such as rockwool.

### Suppression of pathogen growth on carriers

**Figure 1:**
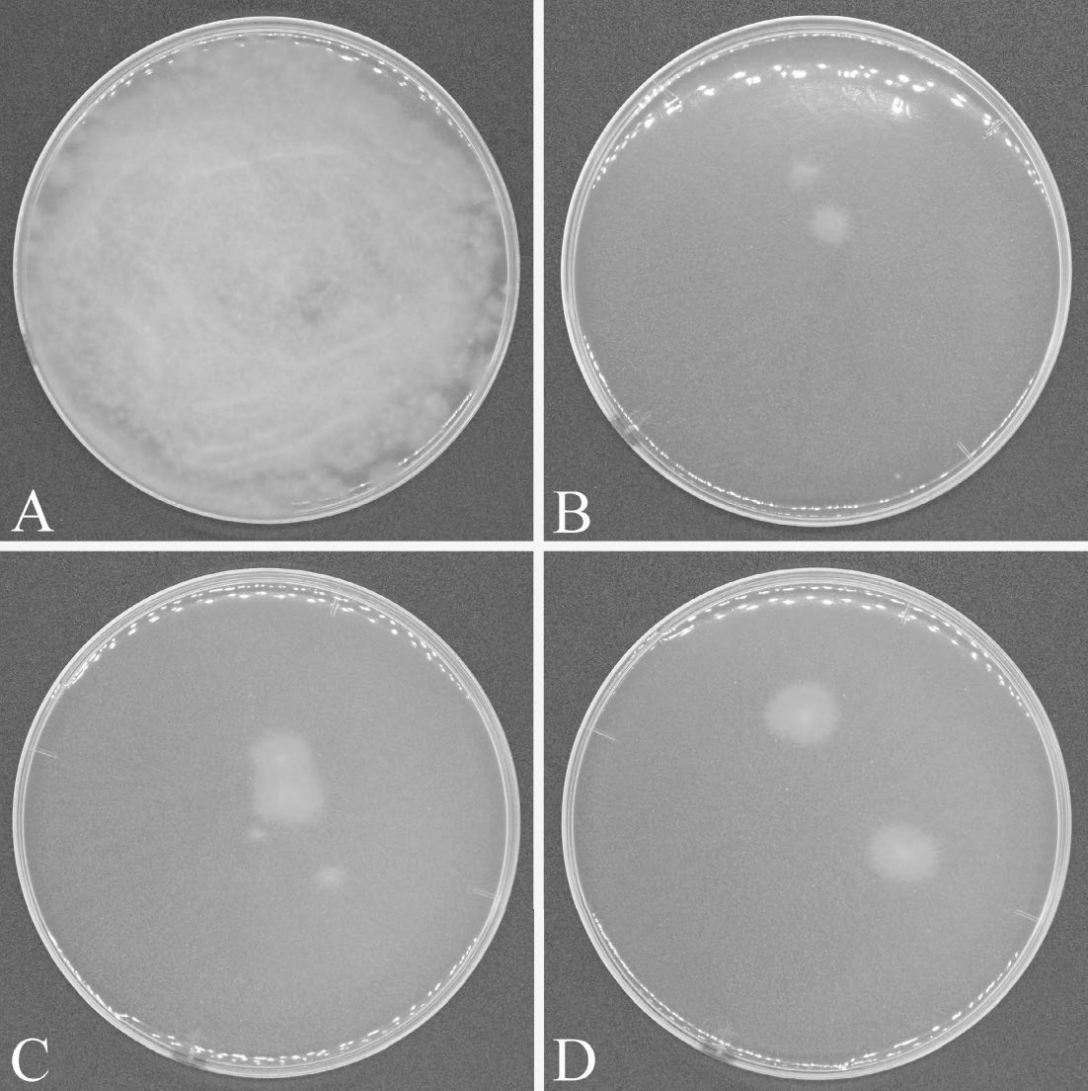
Suppression of *Fusarium oxysporum* f. sp. *lactucae* J. C. Hubb. & Gerik strain H111 growth on non-soil carriers: **(A)** rockwool control with only 1 g/L fish-based soluble fertilizer added; **(B–D)** microorganisms from the MPM solution immobilized on **(B)** rockwool, **(C)** rice husk charcoal, **(D)** vermiculite.

The growth of *Fusarium* was suppressed on rice husk charcoal, rockwool and vermiculite inoculated with MPM solution (Fig. 1, Table 1), to only 10^2^ cfu/mL, but was supported in the controls, with 1 g/L fish-based soluble fertilizer and carriers, to > 10^6^ cfu/mL.

**Table 1:** Suppression of pathogen growth on carriers.

Nutrient sources	*Fusarium oxysporum* f. sp. *lactucae* J. C. Hubb. & Gerik on the non-soil carrier (cfu/mL)		
	Rice hush charcoal	Rockwool	Vermiculite
MPM	0.3 × 10^2^	3.3 × 10^2^	0.2 × 10^2^
1 g/L fish-based soluble (control)	2.9 × 10^6^	1.3 × 10^6^	5.3 × 10^6^

### Suppression of pathogen growth by fertilizers

**Figure 2:**
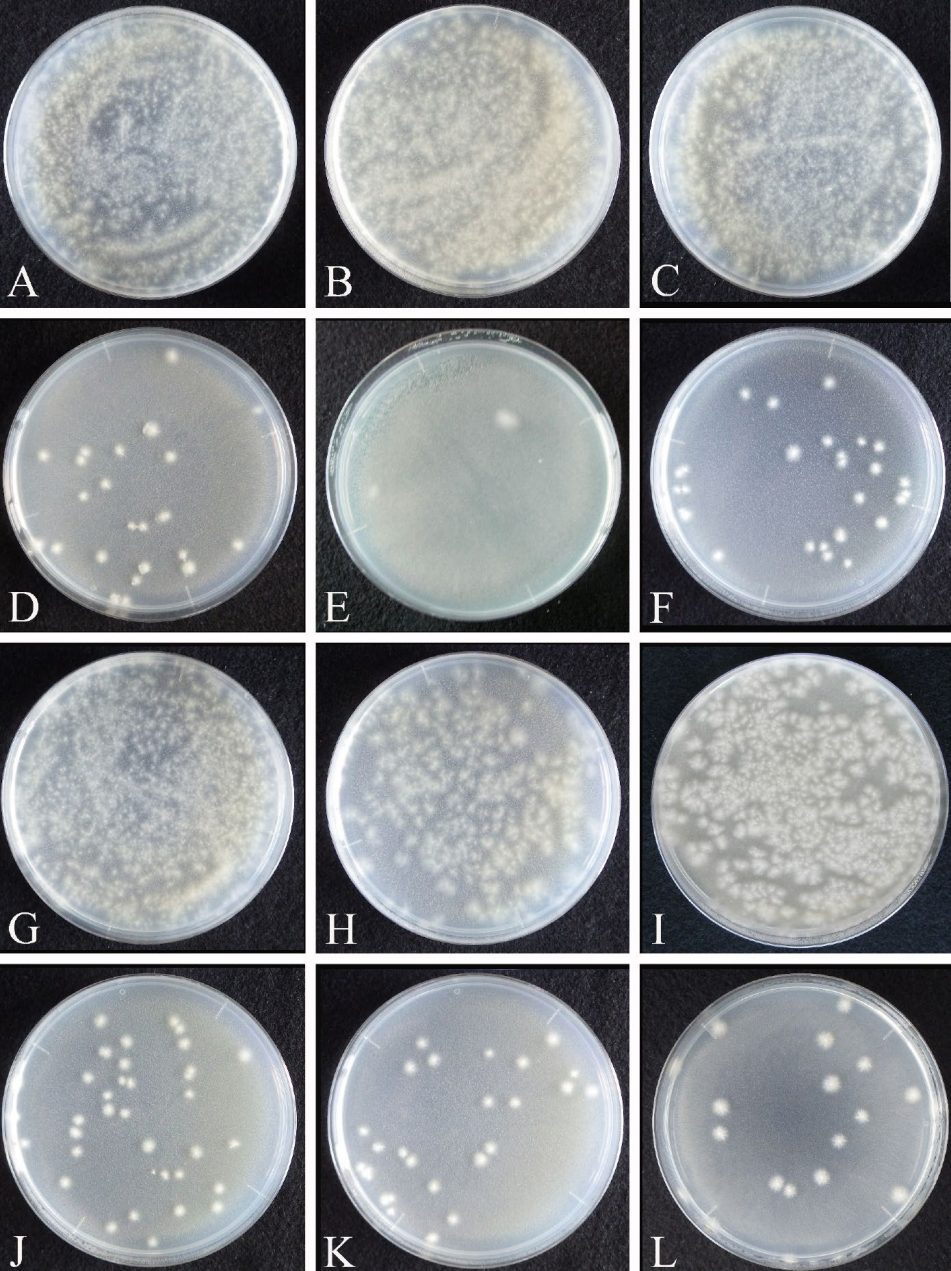
Suppression of *Fusarium oxysporum* f. sp*. lactucae* J. C. Hubb. & Gerik strain H111 on rockwool with different fertilizers. Controls: **(A)** 1 g/L corn steep solids, **(B)** 1 g/L fish-based soluble fertilizer, **(C)** 1 g/L nutrient broth, **(G)** 1 g/L peptone, **(H)** 1 g/L tryptone, and **(I)** 1 g/L yeast extract. Treatments (with MPM microorganisms immobilized on rockwool): **(D)** 1 g/L corn steep solids, **(E)** 1 g/L fish-based soluble fertilizer, **(F)** 1 g/L nutrient broth, **(J)** 1 g/L peptone, **(K)** 1 g/L tryptone, and **(L)** 1 g/L yeast extract.

In rockwool incubated with all nutrient sources (corn steep solids, fish-based soluble fertilizer, nutrient broth, peptone, tryptone, and yeast extract) inoculated with MPM solution, the growth of *F. oxysporum* f. sp. *lactucae* J. C. Hubb. & Gerik was suppressed to a low density regardless of organic fertilizer (Fig. 2, Table 2). The density of *F. oxysporum* f. sp. *lactucae* J. C. Hubb. & Gerik was about 10^2^ cfu/mL with fish-based soluble fertilizer and about 10^3^ cfu/mL with corn steep solids, nutrient broth, peptone, tryptone, and yeast extract, but values reached more than 10^6^ cfu/mL in the control for each fertilizer (Table 2).

**Table 2:** Suppression of pathogen growth by fertilizers.

Nutrient sources	*Fusarium oxysporum* f. sp. *lactucae* J. C. Hubb. & Gerik on the various fertilizers (cfu/mL)
1. MPM with corn steep solids	2.4 × 10^3^
Corn steep solids (control)	3.3 × 10^6^
2. MPM with fish-based soluble	4.7 × 10^2^
Fish based soluble (control)	1.7 × 10^6^
3. MPM with nutrient broth	2.9 × 10^3^
Nutrient broth (control)	3.5 × 10^6^
4. MPM with peptone	3.5 × 10^3^
Peptone (control)	2.8 × 10^6^
5. MPM with tryptone	2.5 × 10^3^
Tryptone (control)	1.9 × 10^6^
6. MPM with yeast extract	1.8 × 10^3^
Yeast extract (control)	3.3 × 10^6^

### Antagonism in plant inoculations

**Figure 3:**
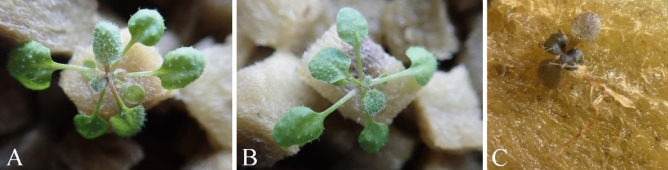
Suppression of *Fusarium oxysporum* f. sp. *conglutinans* (Wollenweber) Snyder et Hansen strain Cong:11 on *Arabidopsis thaliana* (L.) Heynh. Treatments: **(A,B)** with the microorganisms from the MPM solution immobilized on rockwool **(A)** without (control) and **(B)** with *F. oxysporum* f. sp. *conglutinans* (Wollenweber) Snyder et Hansen strain Cong:11 inoculum; **(C)** without the microorganisms from the MPM solution but with inorganic fertilizer and *F. oxysporum* f. sp. *conglutinans* (Wollenweber) Snyder et Hansen strain Cong:11 inoculum.

**Figure 4:**
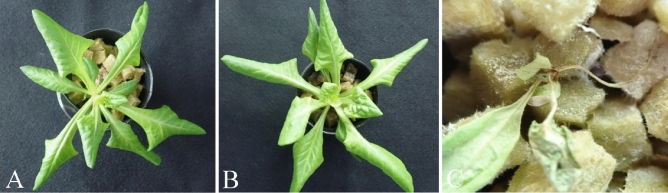
Suppression of *Fusarium oxysporum* f. sp. *lactucae* J. C. Hubb. & Gerik on *Lactuca sativa* L. var. *capitata*. Treatments: **(A,B)** with the microorganisms from the MPM solution immobilized on rockwool **(A)** without (control) and **(B)** with *F. oxysporum* f. sp. *lactucae* J. C. Hubb. & Gerik inoculum; **(C)** without the microorganisms from the MPM solution but with inorganic fertilizer and *F. oxysporum* f. sp. *lactucae* J. C. Hubb. & Gerik inoculum.

*Arabidopsis thaliana* (L.) Heynh. and *L. sativa* seedlings grown with MPM solution organisms immobilized on rockwool showed no wilting symptoms at 10 days after inoculation in the uninoculated control (Figs. 3A, 4A) or when they were inoculated with *F. oxysporum* f. sp. *conglutinans* (Wollenweber) Snyder et Hansen strain Cong:11 and *F. oxysporum* f. sp. *lactucae* J. C. Hubb. & Gerik (Figs. 3B, 4B), whereas inoculated seedlings grown in the inorganic nutrients without the MPM microorganisms showed severe wilting (Figs. 3C, 4C).

## Discussion

In soil-less culture, non-soil media such as rice husk charcoal, rockwool, and vermiculite are used instead of soil, but they do not contain a microbial ecosystem that performs the same function as microorganisms in soil (i.e., degrading organic matter into inorganic nutrients). Conventional hydroponics is limited by the inability to use organic fertilizer in the solution; if organic fertilizers are added, the solution becomes turbid and the plant roots become damaged^6,14^ . If solid media are used in hydroponics, they must be able to degrade organic fertilizers by immobilizing the microbial ecosystem. The hydroponic techniques developed by Shinohara et al. (2011) can use organic fertilizer (because the media are capable of degrading the organic fertilizer into inorganic forms) and can also lower the incidence of root rot^2,3,7^.

Here, we tested the microbial ecosystem that became established and was immobilized on non-soil carriers for its ability to suppress disease while still promoting plant growth. Our method inhibited root diseases in the model hydroponic system. This technique therefore has the potential to control *Fusarium* wilt and other root diseases in hydroponic systems using non-soil carriers. Suppression of root diseases, such as bacterial wilt disease in tomato and *Fusarium* root rot in tomato and lettuce, has been observed in organic hydroponics systems only without solid media ^7^. Here, we inoculated the phytopathogenic fungus *F. oxysporum* f. sp. *lactucae* J. C. Hubb. & Gerik in media in which a microbial ecosystem from MPM culture solution was immobilized on a non-soil carrier. This system clearly inhibited the growth of *F. oxysporum* f. sp. *lactucae* J. C. Hubb. & Gerik and its ability to cause disease (Figs. 1, 2 and 4). The results indicate that the disease-suppressive effect can be reproduced in a non-soil carrier. The microbial ecosystem that developed here had abilities similar to that of soil to degrade organic fertilizer into inorganic nutrients and to suppress root disease. The inoculation of MPM microorganisms can therefore be an eco-friendly method to control *Fusarium* wilt and other root diseases in hydroponic systems, although it will be necessary to test it to determine its effectiveness against other pathogens. In addition, the MPM microbial ecosystem contains more than 50,000 microbes^8^, making it difficult to identify the relationship between disease suppression and specific components of the microbial ecosystem. It will be necessary to see whether it is possible to reproduce the observed disease control in a microbial ecosystem composed of a smaller number of microorganisms, as this would facilitate its implementation on an operational scale.

## Data Availability

All data generated or analysed during this study are included in this published article.
